# PP70 Privacy And Confidentiality In Health Monitoring For Elderly People Using Wearable Electronic Devices: A Scoping Review

**DOI:** 10.1017/S0266462325102882

**Published:** 2025-12-29

**Authors:** Alan Martinez Kozyreff, Lívia Luize Marengo, Cristiane De Cassia Bergamaschi Motta, Fabio da Silva Moraes, Laura Inês Maricato Otani, Silvio Barberato-Filho

## Abstract

**Introduction:**

Monitoring elderly health via wearable electronic devices offers significant benefits but faces ethical and regulatory challenges. While these devices are useful in emergencies, such as falls, concerns about user privacy and data confidentiality persist. This study aimed to develop recommendations for developers of wearable electronic devices used in elderly health monitoring, as well as for other stakeholders, focusing on privacy and confidentiality.

**Methods:**

A scoping review was performed by searching databases including MEDLINE (PubMed), Embase, Scopus, the Cochrane Library, Web of Science, Epistemonikos, and the Virtual Health Library through to October 2023. Studies were included if they addressed all the following criteria: (i) wearable electronic devices; (ii) elderly individuals; (iii) health monitoring; and (iv) privacy, confidentiality, or data protection. Studies that did not include recommendations for developers or other stakeholders aimed at enhancing user privacy protection and data confidentiality were excluded. The results were presented as a narrative synthesis.

**Results:**

A total of 1,037 records were identified in the databases, and 12 studies published between 2014 and 2022 were included. The main findings were addressed in topics, including: (i) privacy and confidentiality in monitoring the health of elderly individuals using wearable electronic devices; (ii) barriers and facilitators for the adoption of wearable devices in monitoring the health of elderly individuals; and (iii) recommendations for developers of wearable devices in monitoring the health of elderly individuals and for other stakeholders.

**Conclusions:**

The use of wearable electronic devices for health monitoring in elderly individuals presents complex ethical, regulatory, and technological challenges, particularly regarding privacy and confidentiality. Collaboration between developers and stakeholders is essential for implementing robust security measures and establishing effective regulatory standards that ensure comprehensive user protection.

